# HIV‐1 DNA decay is faster in children who initiate ART shortly after birth than later

**DOI:** 10.1002/jia2.25368

**Published:** 2019-08-23

**Authors:** Kirsten A Veldsman, Anita Janse van Rensburg, Shahieda Isaacs, Shalena Naidoo, Barbara Laughton, Carl Lombard, Mark F Cotton, John W Mellors, Gert U van Zyl

**Affiliations:** ^1^ Division of Medical Virology Stellenbosch University Faculty of Medicine and Health Sciences Cape Town South Africa; ^2^ Department Pediatrics and Child Health Tygerberg Children's Hospital and Family Clinical Research Unit Stellenbosch University Cape Town South Africa; ^3^ Division of Epidemiology and Biostatistics Department of Global Health Faculty of Medicine and Health Sciences Stellenbosch University Cape Town South Africa; ^4^ Division of Infectious Diseases Department of Medicine University of Pittsburgh Pittsburgh PA USA; ^5^ National Health Laboratory Service Cape Town South Africa; ^6^ Biostatistics Unit South African Medical Research Council Cape Town South Africa

**Keywords:** HIV‐1 DNA kinetics, early treatment, early infant HIV‐1 diagnosis, HIV‐1 persistence, paediatrics, Africa

## Abstract

**Introduction:**

There is limited data in children on whether persistence of HIV‐1 infected cells is affected by age at initiating antiretroviral therapy (ART), its duration or any subsequent ART interruption. We therefore investigated the effects of both age of ART initiation and duration of ART interruption on HIV‐1 DNA decay in children.

**Methods:**

We investigated HIV‐1 DNA decay in three groups of children on ART: Group**‐**1 (n = 7) started uninterrupted ART within eight days of life; Group‐2 (n = 8) started uninterrupted ART at a median of five months of age; and Group‐3 (n = 23) started ART at a median age of 1.8 months for either 40 or 96 weeks, then interrupted ART (median of seven months), and restarted ART based on CD4 count and clinical criteria. Total HIV‐1 DNA was assayed using a sensitive HIV‐1 subtype C‐adapted quantitative PCR for *integrase*. The duration of ART was square root transformed to fit the observed slowing of HIV‐1 DNA decay rate. For each group, point estimates for decay rates were determined after six months of continuous suppressive ART in groups 1 and 2 or six months after restarting ART in Group‐3. Groups‐2 and 3 were combined using a mixed effect regression model to investigate covariates of HIV‐1 DNA decay rate.

**Results and Discussion:**

At six months of continuous suppressive ART, the HIV‐1 DNA t½ (95% CI) was shorter in Group‐1 (n = 7): 2.7 months (2.1 to 3.8), than 9.2 months (7.4 to 12.1) in Group‐2 (n = 8); and 9.6 months (7.6 to 12.6) in Group‐3 (n = 23) (*p *<* *0.01). In multivariable analyses, HIV‐1 DNA before treatment (*p *<* *0.001) and the change in HIV‐1 DNA during interruption (*p *<* *0.01) were independent predictors of slower HIV‐1 DNA decay.

**Conclusions:**

These data suggest that ART initiation within the first week of life can reduce the persistence of long‐lived infected cells. Delaying ART is associated with slower decay of infected cells.

## 1 Introduction

ART initiation during early stages of HIV‐1 infection, Fiebig I‐IV, in adults 1 and in the first six months of life in children 2 is associated with lower levels of persistent HIV‐1 infected cells. Some individuals, such as the Mississippi baby 3, who started ART at 30 hours of life, and adults who started ART between 10 and 12 days after infection 4, had delayed viral rebound after stopping ART, which is most likely due to a smaller pool of replication‐competent proviruses that could stochastically reactivate 5. Early therapy is also associated with an increased probability of post‐treatment control in adults 6, 7, and in two children 8, 9. A cross‐sectional study of South African children revealed lower HIV‐1 total DNA levels in those starting ART before two months‐of‐age compared to later 10. There are limited data in children, however, on the influence of age at ART initiation and respective duration of initial ART and ART interruption on the levels of HIV‐1 infected cells or their longitudinal decay. In a recent study of adults with an ART interruption for a median of 57 days, restarted when plasma HIV‐1 RNA was >1000 copies/mL, for more than two weeks, there was no difference in intact HIV‐1 sequences before and after interruption 11.

The Children with Early Antiretroviral Therapy (CHER) trial randomized children to early, time‐limited ART or delayed continuous ART. Children were enrolled into the CHER study between August 2005 and December 2007 12 when children were first diagnosed between four and six weeks of age, representing a mixture of perinatal and intra‐uterine HIV‐1 infection. As the risk‐benefit of early versus delayed ART in perinatally infected children had not been known, children from the CHER trial were randomized to elective early time‐limited ART: either 40 (ART‐40W) or 96 weeks (ART‐96W) of initial treatment, or continuous therapy, deferred until clinical progression or CD4 decline meeting concurrent ART start criteria (ART‐Def) 13. Children, whose ART was interrupted, reinitiated ART based on the same CD4 or clinical criteria, resulting in variable periods off ART. All interrupted participants except one, from another site, met reinitiation criteria 8. Despite a period of ART interruption, the CHER study showed that early elective ART had clinical and immunological benefit over delayed uninterrupted ART after a median follow‐up period of 4.8 years on study 13. Subsequently, ART programmes introduced infant HIV‐1 PCR testing followed by immediate ART initiation to avert rapid disease progression and early mortality 12, 14, 15. The impact of this very early treatment strategy on the long‐term persistence of HIV‐1‐infected cells is unknown. Accordingly, the first aim of the current study was to compare the HIV‐1 decay rate in three Groups of children: Group‐1 who started continuous ART as soon as possible after birth 16, to two groups from the CHER trial: Group‐2 started continuous ART at a median of five months of age; Group‐3 initiated ART at a median of 1.8 months of age for either 40 or 96 weeks, and then interrupted ART for median of seven months, restarting ART based on CD4 + T cell count criteria. The second aim was to describe factors associated with the rate of HIV‐1 DNA decay in the CHER participants.

## 2 Methods

### 2.1 Participant selection

Parents or legal guardians provided written informed consent for all participants. The study was approved by the Stellenbosch University Health Research Ethics Committee: M14/07/029.

Children included in the CHER trial primary endpoint analysis which was time‐to‐failure of first‐line ART (immunological/clinical/virological) or death had a CD4 percentage ≥25% at baseline. A small group with a baseline CD4% of <25% at screening, referred to as part B, commenced early ART, had samples stored and were retained in follow‐up. Although some of them initially interrupted ART, the data safety monitoring board subsequently recommended that all Part B remain on continuous ART. Some children, who were randomized to time limited therapy (ART‐96W), were not interrupted as they had already met a trial endpoint. Although plasma HIV‐1 RNA was not measured during interruption, plasma samples were stored and some were available for retrospective plasma HIV‐1 RNA testing. To compare data from interrupted and never‐interrupted participants, HIV‐1 DNA decay was assessed only during “continued” phase ART, defined as therapy initiation in never‐interrupted patients or reinitiation after protocol‐defined treatment interruption. All participants in the present study had at least one sample before continuous ART, that is, before treatment in never‐interrupted children and before reinitiation in those interrupted. The following time points were included to study HIV‐1 DNA kinetics: a baseline (pre‐ART sample in never‐interrupted children, or a sample during protocol‐defined ART interruption, prior to reinitiating ART). Once back on ART, samples were collected closest to the following time points: six, twelve and eighteen months, during the trial. Thereafter samples were collected between two to three years and/or four to five years later. To assess HIV‐1 decay kinetics in the absence of viraemia, participants were censored after the first of two viraemic episodes (plasma HIV‐1 RNA >200 copies/mL) or excluded if less than two samples (including baseline) were collected before viraemia. Based on these criteria, 31 participants were included. Of these, eight individuals were never interrupted (Group‐2), six because they were already in ART‐Def and started a few months later and two randomized to ART‐96W who had already met a trial endpoint. Twenty‐three participants were interrupted based on randomization (Group‐3).

HIV‐1 DNA decay from children in the CHER study was compared to that of seven infants who initiated ART within eight days of life (Group‐1). These children were diagnosed through a public sector birth diagnosis and early treatment initiation programme. Inclusion in this study was based on virological suppression defined as continuous downward trend in plasma HIV‐1 RNA and no HIV‐1 RNA >100 copies/mL at the first measurement after six months on ART 17.

### 2.2 Sample processing and laboratory assays

Samples were processed and stored according to the HIV/AIDS Network Coordination (HANC) peripheral blood mononuclear cell (PBMC) processing standard operating procedure (https://www.hanc.info/labs/labresources/procedures/Pages/pbmcSop.aspx).

HIV‐1 total DNA was extracted and measured by a sensitive quantitative PCR adapted for HIV‐1 subtype C, targeting a conserved region in HIV‐1 integrase (limit of detection: 3 copies/reaction), as previously described 16, 18, 19.

Plasma HIV‐1 RNA monitoring was initially performed with the Roche Amplicor HIV Monitor assay version 1.0 (lower limit of detection (LOD) of 400 copies/mL), then with the Roche Amplicor HIV Monitor assay ultrasensitive protocol (LOD of 50 copies/mL). After completion of the CHER study in August 2011, the Abbott Diagnostics Realtime HIV‐1 assay was used (LOD of 150 copies/mL for 200 μL input or 40 copies/mL for 1.0 mL input). The Roche CAP/CTM v2.0 assay (LOD of 100 copies/mL for 200 μL input or 20 copies/mL for 1 mL input) was used to assess plasma HIV‐1 RNA in the seven infants initiated shortly after birth.

### 2.3 Statistics and modelling

Statistical analyses were performed and graphics were generated using R 3.4.3 20. Because the rate of log_10_ HIV‐1 DNA decline decreased with the time on ART, a square root transformation of time on ART improved the fit of the decay model as determined by conditional R^2^ 21. This model allowed exploring factors affecting decay over the full observed period, rather in separate phases. Viraemia‐copy‐years as cumulative measure of viraemia, during the period of interruption, was calculated as the product of the level of viraemia (log_10_ HIV‐1 RNA copies/mL) multiplied by the duration of viraemia.

Separate mixed effect models with time treated as fixed effect and participant as random effect (random intercepts only) were used to describe decay of HIV‐1 DNA in each of the three groups. As the decay rate decreased the longer participants were treated, the first derivative of the decay curve was used for estimates of HIV‐1 DNA decay rate at six months on continued ART to compare HIV‐1 DNA decay between groups. Wilcoxon Rank Sum Tests were used to assess differences between groups: Linear models (log_10_ HIV‐1 DNA change against the square root of days treated) were fitted for each individual in each group; endpoint HIV‐1 DNA was compared between Groups‐2 and 3.

To assess which factors were independent predictors of HIV‐1 DNA decay among the 31 CHER participants from the point of continued treatment, the following variables with *a priori* evidence of possible association with HIV‐1 DNA decay, as fixed effects, were included in a full model: baseline HIV‐1 DNA, pre‐therapy HIV‐1 plasma RNA and time‐interrupted; participant was included as random effect (independent random intercepts and slopes with linear time effects).

## 3 Results

The baseline and clinical characteristics of the patients included are shown in Table 1.

**Table 1:** Participant characteristics

Participant characteristic	Group‐1: Early continued ART (n = 7)	Group‐2: Suppressed viraemia and continued ART (n = 8)	Group‐3: Suppressed viraemia and interrupted (n = 23)
Treatment regimen	ABC, 3TC, LPV/r^a^	AZT, 3TC, LPV/r	AZT, 3TC, LPV/r
CHER Study arms	NA	ART‐Def (n = 6) ART‐96W (n = 2)^b^	ART‐40W (n = 15) ART‐96W (n = 6) Part B ART‐40W (n = 2)^c^
Age ART first initiated (days); median (IQR)	5 (1.5 to 6.5)	156.5 (110.3 to 256.8)	55 (50.5 to 64.5)
Pre‐treatment log 10 HIV‐1 RNA load; median (IQR)	3.1 (2.7 to 3.3)	5.6 (5.3 to 5.9)	5.3 (4.0 to 5.8)
Baseline ART HIV‐1 DNA copies/million cells; median (IQR)	158 (40 to 398)	1107 (468 to 2999)	832 (363 to 1371)
CD4% nadir^d^; median (IQR)	40 (38.7 to 54.5)	16.9 (14.0 to 19.1)	21.8 (15.9 to 25.2)
Absolute CD4 count nadir cells/microlitre^d^; median (IQR)	1955 (1193 to 2064)	505 (440.5 to 759.5)	871 (577.5 to 1081.5)
Time interrupted (days); median (IQR)	‐	‐	214 (141 to 284)
Age at last sample (years); median (IQR)	1.0 (0.6 to 1.0)	8.8 (1.3 to 9.7)	9.2 (7.8 to 10.6)
CD4% (IQR) at last sample	34 (28.5 to 37.5)	34 (31 to 42)	37 (35 to 39)
Absolute CD4 (IQR) (per microlitre) at last sample	1920 (1440 to 2618)	1032 (814 to 1199)	1088 (847 to 1294)

ART, antiretroviral therapy; CHER, Children with HIV early antiretroviral therapy trial; IQR, interquartile range.

^a^Original regimen was AZT, 3TC, NVP, with NVP replaced by LPV/r at 42 weeks of age and AZT replaced by ABC at 3 months of age; ^b^group‐2 included 2 ART‐96W participants who were not interrupted as both had signs suggestive of HIV encephalopathy; ^c^part B of CHER had screening CD4 <25%; ^d^before continued phase of treatment.

Three groups of children were compared: Group‐1 comprised seven infants who started continued ART within eight days of birth; median 5 (IQR: 0 to 7) days, Group‐2 comprised eight children who started continuous ART at a median of 5.1 (IQR: 3.6 to 8.4) months, and Group‐3 comprised 23 children who started ART at a median of 1.8 (IQR: 1.7 to 2.1) months but with ART interruption for a median of 7 (IQR: 4.6 to 9.3) months and who reinitiated at a median age of 20.2 (IQR: 16.0 to 30.9) months.

### 3.1 Endpoint HIV‐1 DNA

In Group‐1, HIV‐1 DNA declined to <10 copies/million cells in six of seven (85.7%) infants at a median of 6.9 months on ART 17. In Group‐2, five of the eight children (62.5%) had samples collected, after at least four years of suppressive ART (median 8.6 years). Here HIV‐1 DNA became undetectable (<3 copies/million cells) in two children, declined to 20.0 copies/million cells in 2, and 39.8 copies/million cells in another child (the remaining three children were censored due to episodes of viraemia before four years on ART). In Group‐3, 18 of the 23 (78.3%) had samples collected after at least four years (after a median of 7.8 years after resuming continuous ART), HIV‐1 DNA became undetectable in 4, and declined to median of 31.6 (range 7.9 to 398.1) copies/million cells in another 14 (the remaining five were censored before four years on ART due to episodes of viraemia). Despite ART interruption in Group‐3 there was no difference in endpoint HIV‐1 DNA at a similar period on treatment between Groups‐2 and 3 (*p *=* *0.5) and no difference in total CD4 count median (IQR) at study end, Group‐2: 1032 (814 to 1199); Group‐3: 1088 (847 to 1294) cells/μl (*p *=* *0.7).

### 3.2 Comparison of HIV‐1 DNA decay rates

**Figure 1:**
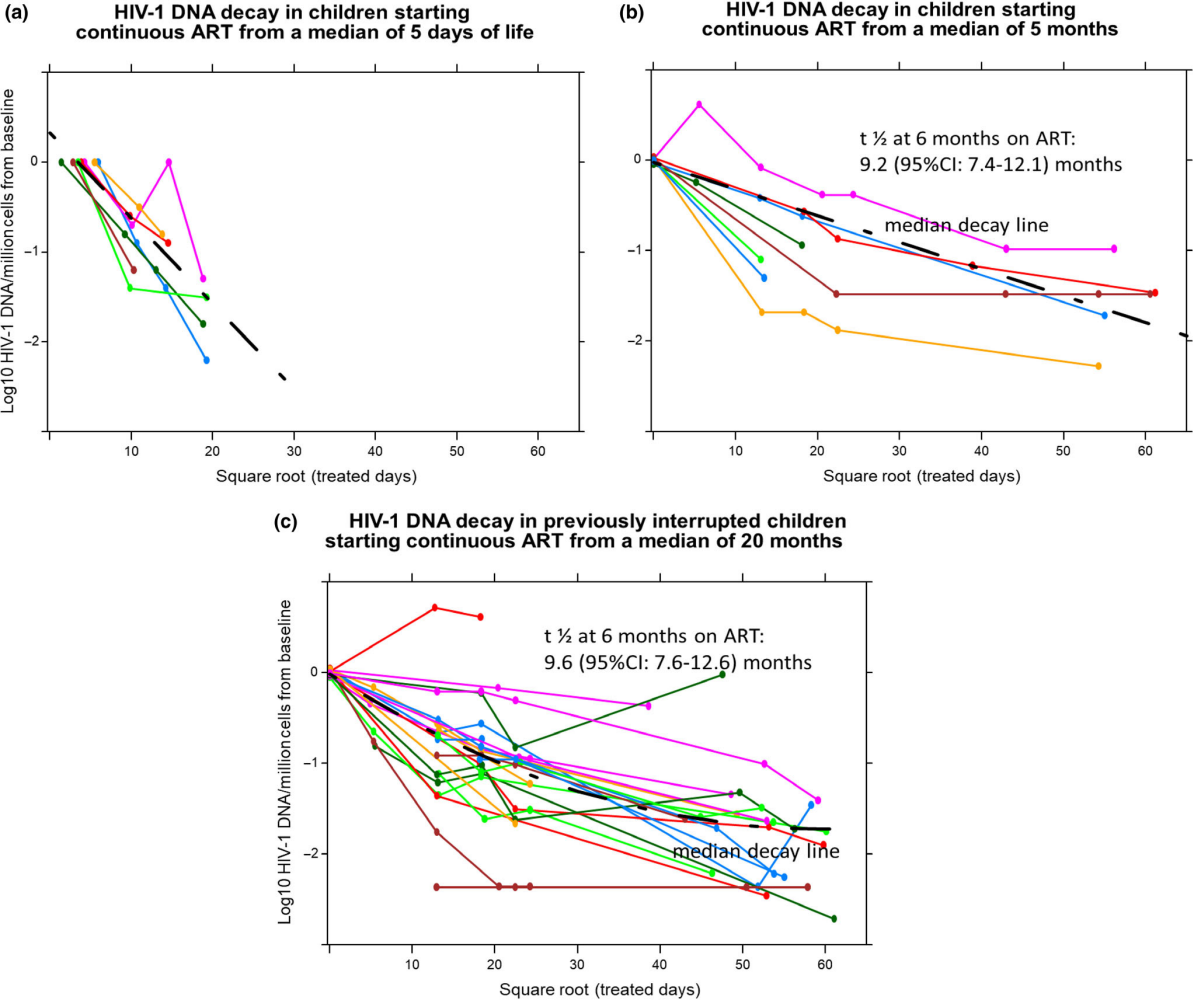
HIV‐1 DNA decay in three groups of children on ART **(a)** Group‐1: Continued ART from before eight days (median five days) (n = 7). **(b)** Group‐2 began continued suppressive ART at a median of five months of age (n = 8). **(c)** Group‐3: Early ART for 40 or 96 weeks from a median of 1.8 months (n = 23); ART interruption for a median of seven months and continued ART from a median age of 20 months. HIV‐1 DNA levels reached levels <10 copies/million cells in six of seven (85.7%) very early treated individuals (Group‐1) at a median of 6.7 months on ART compared to three of five (60%) of never‐interrupted children starting later, and five of eighteen (28%) of interrupted children, after a median of eight to nine years on treatment.

The conditional R^2^ (95% CI) values for the model HIV‐1 DNA decay estimates were 0.82 (0.65 to 0.93) for Group‐1 (early start), 0.85 (0.67 to 0.94) for Group‐2 (later start) and 0.79 (0.68 to 0.86) for Group‐3 (interrupted), indicating an overall good model fit. The t ½ of HIV‐1 DNA in Group‐1 at six months of continuous treatment was 2.7 (95% CI: 2.1 to 3.8) months, being significantly shorter than both the eight children from Group‐2 at 9.2 months (95% CI: 7.4 to 12.1; *p *<* *0.01), and the 23 children from Group‐3 (9.6 months; 95% CI: 7.6 to 12.6 months; *p *<* *0.01) (Figure 1). Decay rates in the latter two groups were similar (*p *=* *0.81).

### 3.3 Associations with HIV‐1 DNA decay rates

Univariate analyses of the 31 CHER children, using a mixed effect model of decay, showed that only pre‐treatment total HIV‐1 DNA (*p *<* *0.001) was a significant predictor of slower decay. Neither age at ART start, nadir CD4 count or nadir CD4%, baseline plasma HIV‐1 RNA, time off ART, viraemia‐copy‐years during interruption, nor original CHER study arm, were significant predictors of the rate of decay.

In multivariable mixed effects models (Table 2), slower decay was associated with HIV‐1 DNA level at baseline (*p *<* *0.0001), faster decay with a higher baseline plasma HIV‐1 RNA load (*p *=* *0.033) and belonging to ART‐96W or ART‐40W (versus ART‐Def) of the CHER study. Time‐interrupted (*p *=* *0.314), was not an independent significant predictor of HIV‐1 DNA decay.

**Table 2:** Mixed effect model of Log_10_ HIV‐1 DNA decay against the square root of time (days treated) for children on continued ART

Fixed effects	Coefficient	SE	Df	t‐value	*p*(>|t|)
(Intercept)	1.332	0.451	19.441	2.952	0.008
Square root of days treated	−0.028	0.002	12.462	−13.689	<0.0001^a^
Baseline HIV‐1 DNA level	0.799	0.115	20.852	6.955	<0.0001
Pre‐treatment HIV‐1 RNA level	−0.159	0.069	19.528	−2.295	0.033
Time interrupted	0.000	0.000	24.036	1.029	0.314
Study arm					
ART‐40W (vs. ART‐Def)	−0.406	0.195	22.631	−2.085	0.049^a^
ART‐96W (vs. ART‐Def)	−0.532	0.204	21.993	−2.606	0.016^a^
Part B ART‐40W (vs. ART‐Def)	0.115	0.312	24.378	0.369	0.715

Groups	Name	Variance	SD
For Random effects			
Participant	(Intercept)	0.078	0.279
Participant	Square root of days treated	0.000015	0.0039
Residual		0.145	0.380

a Significant *p*‐values (*p *<* *0.05); CHER part A were randomized to delayed continuous therapy (ART‐Def) or time limited early elective therapy for 40 or 96 weeks respectively (ART‐40W or ART‐96W); Part B had a baseline CD4% <25% and received initial therapy for 40 weeks. The coefficients of the first four fixed effects factors represent the change in log10 (HIV‐1 DNA) for a one unit change in the factor adjusted for the other factors in the model. When assessing the association with HIV‐1 DNA decay and using the t‐statistic (a standardized difference) the days treated and baseline HIV‐1 DNA level have a strong effect on slowing decay, whereas pre‐treatment HIV‐1 RNA level has a small effect on increasing decay. The effect of time interrupted is not significant. With reference to both ART‐Def, ART‐96W and ART‐40W has a small effect, increasing decay, whereas the effect of Part B ART‐40W on decay was not significant. The difference in coefficients for ART‐40W versus 96W was not significant (*p *=* *0.68), but the overall effect of treatment arm was significant (*p *=* *0.037). The variance components for random intercepts, slopes and error are presented and in general, they are small and estimated with some uncertainty given the standard deviation of these estimates.

## 4 Discussion

The decay of HIV‐1 DNA was much faster in children diagnosed at birth who started ART at a median of 5 days of life (Group‐1) than those starting several weeks later, Groups‐2 and 3, with no difference between the latter groups. In Groups‐2 and 3 it is uncertain whether infection occurred *in utero* or intra‐partum, which could have influenced decay rate, as *in utero* infection had time to become more established. In Groups‐2 and 3, the strongest predictors of a slower decay rate was a higher pre‐treatment HIV‐1 DNA level. The median age of ART initiation was later in Group‐2 (uninterrupted) than in Group‐3 (interrupted), who reinitiated ART based on CD4 and clinical criteria. Therefore, those with more rapid deterioration during interruption were initiated faster. These factors could have counteracted any seeding of infected cells during interruption or effect of earlier ART.

Very early therapy may prevent the seeding of long‐lived reservoirs such as CD4 clones that harbour replication competent virus 22. Other studies found lower HIV‐1 DNA levels in children who started ART before three months 19, 23, 24, 25, six months 2 or one year 26 versus later, and a positive association between the timing of starting therapy and better viraemia suppression and lower total HIV‐1 DNA after four to eleven years on treatment 27. There are few studies in children that describe longitudinal HIV‐1 DNA data. One such study found no difference in the rate of decay in children starting ART <3 months or later 24. Taken together, this suggests that there is a very early window when the pool of HIV‐1 infected cells is more labile and when ART may reduce the seeding and persistence of long‐surviving reservoir cells. No child in this study received integrase strand transfer inhibitors (INSTI) which might have resulted in faster HIV‐1 DNA decay than in the current study 28, 29.

The limitations of this study included the following: We had longitudinal samples from only seven children treated shortly after birth, none of whom had PBMC samples collected prior to therapy initiation. In addition, we had access to longitudinal samples of only 31 individuals with suppressed viraemia from the CHER study, a larger sample size may have enabled us to detect a smaller effect of treatment interruption on HIV‐1 DNA decay. Moreover, although HIV‐1 DNA levels decayed to undetectable levels in a number of children, this does not indicate the absence of persisting replication‐competent reservoirs. Children in CHER were reinitiated based on CD4 and clinical criteria. The current norm for studies that involve therapy interruption is to perform regular plasma HIV‐1 RNA, immunologic and clinical safety monitoring to decide when to reinitiate therapy, referred to as intensively monitored antiretroviral therapy pause (IMAP). Nevertheless, there was no statistical difference in the decay rates and endpoint HIV‐1 DNA between interrupted and uninterrupted children in the participants from CHER, but any affect could have been obscured by a later start in participants who did not interrupt ART.

## 5 Conclusions

Birth diagnosis followed by early treatment initiation before eight days of life was associated with a significantly faster decay of HIV‐1‐infected cells compared to those tested around four to six weeks of life and then receiving early time‐limited ART or delayed continuous ART. Early elective treatment initiation and a sufficiently long duration of initial ART may limit the seeding of long‐surviving HIV‐1 infected cells, and impact health and quality of life outcomes. Due to their small pools of HIV‐1 infected cells and largely intact immune responses, early treated individuals may be valuable candidates for functional cure studies. There is a need for studies of very early treated children that involve intensive monitoring during periods of interruption to investigate safety and the impact of interruption on the HIV‐1 reservoir.

## Competing interests

GvZ has received an honorarium for a scientific presentation from ViiV health not related to the current work. JWM is a paid consultant to Gilead Sciences and Merck and owns share options in Co‐Crystal Pharma, Inc.

## Authors’ contributions

GvZ drafted the manuscript and performed analyses with advice from CL. KAV performed the HIV‐1 DNA assays and summarized the laboratory results. SI and SN were responsible for the laboratory study database and processing of samples. AvR recorded clinical data and recruited patients. BL and MFC were responsible for clinical oversight. JWM and MFC provided scientific guidance. All authors reviewed and accepted the final manuscript.

## References

[1] Ananworanich J , Chomont N , Eller LA , Kroon E , Tovanabutra S , Bose M , et al. HIV DNA set point is rapidly established in acute HIV infection and dramatically reduced by early ART. EBioMedicine [Internet]. 2016 [cited 2017 Jan 13];11:68–72. Available from: http://www.ncbi.nlm.nih.gov/pubmed/27460436 27460436 10.1016/j.ebiom.2016.07.024 PMC5049918

[2] Ananworanich J , Puthanakit T , Suntarattiwong P , Chokephaibulkit K , Kerr SJ , Fromentin R , et al. Reduced markers of HIV persistence and restricted HIV‐specific immune responses after early antiretroviral therapy in children. AIDS [Internet]. 2014 [cited 2017 Jan 13];28:1015–20. Available from: http://content.wkhealth.com/linkback/openurl?sid=WKPTLP:landingpage&an=00002030-201404240-00010 24384692 10.1097/QAD.0000000000000178

[3] Luzuriaga K , Gay H , Ziemniak C , Sanborn KB , Somasundaran M , Rainwater‐Lovett K , et al. Viremic relapse after HIV‐1 remission in a perinatally infected child. N Engl J Med [Internet]. 2015 [cited 2016 Nov 25];372:786–8. Available from: http://www.nejm.org/doi/abs/10.1056/NEJMc1413931 25693029 10.1056/NEJMc1413931 PMC4440331

[4] Henrich TJ , Hatano H , Bacon O , Hogan LE , Rutishauser R , Hill A , et al. HIV‐1 persistence following extremely early initiation of antiretroviral therapy (ART) during acute HIV‐1 infection: An observational study. Bekker L‐G, editor. PLoS Med [Internet]. 2017 [cited 2018 Aug 8];14:e1002417 Available from: http://www.ncbi.nlm.nih.gov/pubmed/29112956 29112956 10.1371/journal.pmed.1002417 PMC5675377

[5] Hill AL , Rosenbloom DIS , Fu F , Nowak MA , Siliciano RF . Predicting the outcomes of treatment to eradicate the latent reservoir for HIV‐1. Proc Natl Acad Sci USA. 2014;111:13475–80. 25097264 10.1073/pnas.1406663111 PMC4169952

[6] Sáez‐Cirión A , Bacchus C , Hocqueloux L , Avettand‐Fenoel V , Girault I , Lecuroux C , et al. Post‐treatment HIV‐1 controllers with a long‐term virological remission after the interruption of early initiated antiretroviral therapy ANRS VISCONTI Study. PLoS Pathog [Internet]. 2013 [cited 2014 Jul 16];9:e1003211 Available from: http://www.pubmedcentral.nih.gov/articlerender.fcgi?artid=3597518&tool=pmcentrez&rendertype=abstract 23516360 10.1371/journal.ppat.1003211 PMC3597518

[7] Martin GE , Gossez M , Williams JP , Stöhr W , Meyerowitz J , Leitman EM , et al. Post‐treatment control or treated controllers? Viral remission in treated and untreated primary HIV infection. AIDS [Internet]. 2017 [cited 2017 Sep 9];31:477–84. Available from: http://www.ncbi.nlm.nih.gov/pubmed/28060012 28060012 10.1097/QAD.0000000000001382 PMC5278888

[8] Violari A , Cotton MF , Kuhn L , Schramm DB , Paximadis M , Loubser S , et al. A child with perinatal HIV infection and long‐term sustained virological control following antiretroviral treatment cessation. Nat Commun [Internet]. 2019 [cited 2019 Feb 4];10:412 Available from: http://www.nature.com/articles/s41467-019-08311-0 30679439 10.1038/s41467-019-08311-0 PMC6345921

[9] Frange P , Faye A , Avettand‐Fenoël V , Bellaton E , Descamps D , Angin M , et al. HIV‐1 virological remission lasting more than 12 years after interruption of early antiretroviral therapy in a perinatally infected teenager enrolled in the French ANRS EPF‐CO10 paediatric cohort: a case report. Lancet HIV [Internet]. 2016 [cited 2017 Sep 16];3:e49–54. Available from: http://www.ncbi.nlm.nih.gov/pubmed/26762993 26762993 10.1016/S2352-3018(15)00232-5

[10] Kuhn L , Paximadis M , Da Costa Dias B , Loubser S , Strehlau R , Patel F , et al. Age at antiretroviral therapy initiation and cell‐associated HIV‐1 DNA levels in HIV‐1‐infected children. Sluis‐Cremer N, editor. PLoS One [Internet]. 2018 [cited 2018 Aug 8];13:e0195514 Available from: http://www.ncbi.nlm.nih.gov/pubmed/29649264 29649264 10.1371/journal.pone.0195514 PMC5896970

[11] Clarridge KE , Blazkova J , Einkauf K , Petrone M , Refsland EW , Justement JS , et al. Effect of analytical treatment interruption and reinitiation of antiretroviral therapy on HIV reservoirs and immunologic parameters in infected individuals. Swanstrom R, editor. PLoS Pathog [Internet]. 2018 [cited 2018 Aug 8];14:e1006792 Available from: http://www.ncbi.nlm.nih.gov/pubmed/29324842 29324842 10.1371/journal.ppat.1006792 PMC5764487

[12] Violari A , Cotton MF , Gibb DM , Babiker AG , Steyn J , Madhi SA , et al. Early antiretroviral therapy and mortality among HIV‐infected infants. N Engl J Med [Internet] 2008 [cited 2018 Nov 21];359:2233–44. Available from: http://www.ncbi.nlm.nih.gov/entrez/query.fcgi?cmd=Retrieve&db=PubMed&dopt=Citation&list_uids=19020325 19020325 10.1056/NEJMoa0800971 PMC2950021

[13] Cotton MF , Violari A , Otwombe K , Panchia R , Dobbels E , Rabie H , et al. Early time‐limited antiretroviral therapy versus deferred therapy in South African infants infected with HIV : results from the children with HIV early antiretroviral (CHER) randomised trial. Lancet. 2013;6736:1–9. 10.1016/S0140-6736(13)61409-9 PMC4104982 24209829

[14] Lilian RR , Kalk E , Technau K‐G , Sherman GG . Birth diagnosis of HIV infection in infants to reduce infant mortality and monitor for elimination of mother‐to‐child transmission. Pediatr Infect Dis J [Internet]. 2013 [cited 2017 Jan 13];32:1080–5. Available from: http://content.wkhealth.com/linkback/openurl?sid=WKPTLP:landingpage&an=00006454-201310000-00012 23574775 10.1097/INF.0b013e318290622e

[15] Bourne DE , Thompson M , Brody LL , Cotton M , Draper B , Laubscher R , et al. Emergence of a peak in early infant mortality due to HIV/AIDS in South Africa. AIDS [Internet]. 2009 [cited 2019 May 24];23:101–6. Available from: https://insights.ovid.com/crossref?an=00002030-200901020-00014 19065753 10.1097/qad.0b013e32831c54bd

[16] Veldsman KA , Maritz J , Isaacs S , Katusiime MG , Janse Van Rensburg A , Laughton B , et al. Rapid decline of HIV‐1 DNA and RNA in infants starting very early antiretroviral therapy may pose a diagnostic challenge. AIDS [Internet]. 2018 [cited 2018 Mar 16];32:629–34. Available from: http://insights.ovid.com/crossref?an=00002030-900000000-97320 29334551 10.1097/QAD.0000000000001739 PMC5834387

[17] Veldsman K , Maritz J , Isaacs S , Katusiime MG , la Grange H , van Rensburg A , et al. Rapid decline of total HIV DNA in children starting ART within 8 days of birth. In: CROI 2017 Conference on Retroviruses and Opportunistic Infections February 13‐16. Seattle, WA; 2017.

[18] Hong F , Aga E , Cillo AR , Yates AL , Besson G , Fyne E , et al. Novel assays for measurement of total cell‐associated HIV‐1 DNA and RNA. J Clin Microbiol [Internet]. 2016 [cited 2016 Oct 6];54:902–11. Available from: http://www.ncbi.nlm.nih.gov/pubmed/26763968 26763968 10.1128/JCM.02904-15 PMC4809955

[19] van Zyl G , Bedison M , Janse van Rensburg A , Laughton B , Cotton M , Mellors J . Early antiretroviral therapy in South African children reduces HIV‐1‐infected cells and cell‐associated HIV‐1 RNA in blood mononuclear Cells. J Infect Dis [Internet]. 2015 [cited 2015 Jan 8];212:39–43. Available from: http://jid.oxfordjournals.org/lookup/doi/10.1093/infdis/jiu827 25538273 10.1093/infdis/jiu827 PMC4542592

[20] R Core Team . R: a language and environment for statistical computing [Internet]. Vienna, Austria; 2018 [cited 2019 Apr 4]. Available from: https://www.r-project.org/

[21] Johnson PCD . Extension of Nakagawa & Schielzeth's *R* ^2^ _GLMM_ to random slopes models. O'Hara RB, editor. Methods Ecol Evol [Internet]. 2014 [cited 2017 Jan 4];5:944–6. Available from: http://doi.wiley.com/10.1111/2041-210X.12225 25810896 10.1111/2041-210X.12225 PMC4368045

[22] Simonetti FR , Sobolewski MD , Fyne E , Shao W , Spindler J , Hattori J , et al. Clonally expanded CD4 ^+^ T cells can produce infectious HIV‐1 *in vivo* . Proc Natl Acad Sci [Internet]. 2016 [cited 2018 Sep 12];113:1883–8. Available from: http://www.ncbi.nlm.nih.gov/pubmed/26858442 26858442 10.1073/pnas.1522675113 PMC4763755

[23] Martínez‐Bonet M , Puertas MC , Fortuny C , Ouchi D , Mellado MJ , Rojo P , et al. Establishment and replenishment of the viral reservoir in perinatally HIV‐1‐infected children initiating very early antiretroviral therapy. Clin Infect Dis [Internet]. 2015 [cited 2018 Aug 8];61:1169–78. Available from: http://www.ncbi.nlm.nih.gov/pubmed/26063721 26063721 10.1093/cid/civ456 PMC4560905

[24] McManus M , Mick E , Hudson R , Mofenson LM , Sullivan JL , Somasundaran M , et al. Early combination antiretroviral therapy limits exposure to HIV‐1 replication and cell‐associated HIV‐1 DNA levels in infants. Sluis‐Cremer N, editor. PLoS One [Internet]. 2016 [cited 2017 Jul 20];11:e0154391 Available from: http://dx.plos.org/10.1371/journal.pone.0154391 27104621 10.1371/journal.pone.0154391 PMC4841514

[25] Luzuriaga K , Tabak B , Garber M , Chen YH , Ziemniak C , McManus MM , et al. HIV type 1 (HIV‐1) proviral reservoirs decay continuously under sustained virologic control in HIV‐1‐infected children who received early treatment. J Infect Dis [Internet]. 2014 [cited 2017 Jan 13];210:1529–38. Available from: http://www.ncbi.nlm.nih.gov/pubmed/24850788 24850788 10.1093/infdis/jiu297 PMC4215073

[26] Foster C , Pace M , Kaye S , Hopkins E , Jones M , Robinson N , et al. Early antiretroviral therapy reduces HIV DNA following perinatal HIV infection. AIDS [Internet]. 2017 [cited 2018 Aug 8];31:1847–51. Available from: http://insights.ovid.com/crossref?an=00002030-201708240-00009 28609403 10.1097/QAD.0000000000001565

[27] Tagarro A , Chan M , Zangari P , Ferns B , Foster C , De Rossi A , et al. Early and highly suppressive antiretroviral therapy are main factors associated with low viral reservoir in european perinatally HIV‐infected children. JAIDS J Acquir Immune Defic Syndr [Internet]. 2018 [cited 2018 Dec 3];79:269–76. Available from: http://insights.ovid.com/crossref?an=00126334-201810010-00019 30211778 10.1097/QAI.0000000000001789 PMC6173292

[28] Cardozo EF , Andrade A , Mellors JW , Kuritzkes DR , Perelson AS , Ribeiro RM . Treatment with integrase inhibitor suggests a new interpretation of HIV RNA decay curves that reveals a subset of cells with slow integration. PLoS Pathog [Internet]. 2017 [cited 2018 Nov 23];13:e1006478 Available from: https://journals.plos.org/plospathogens/article?id=10.1371/journal.ppat.1006478 28678879 10.1371/journal.ppat.1006478 PMC5513547

[29] Andrade A , Rosenkranz SL , Cillo AR , Lu D , Daar ES , Jacobson JM , et al. Three distinct phases of HIV‐1 RNA decay in treatment‐naive patients receiving raltegravir‐based antiretroviral therapy: ACTG A5248. J Infect Dis [Internet]. 2013 [cited 2019 Jan 16];208:884–91. Available from: https://academic.oup.com/jid/article-abstract/208/6/884/833010 23801609 10.1093/infdis/jit272 PMC3749011

